# Diurnal Variation of Short-Term Repetitive Maximal Performance and Psychological Variables in Elite Judo Athletes

**DOI:** 10.3389/fphys.2018.01499

**Published:** 2018-10-26

**Authors:** Hamdi Chtourou, Florian Azad Engel, Hassen Fakhfakh, Hazem Fakhfakh, Omar Hammouda, Achraf Ammar, Khaled Trabelsi, Nizar Souissi, Billy Sperlich

**Affiliations:** ^1^UR15JS01: Education, Motricité, Sport et Santé (EM2S), Higher Institute of Sport and Physical Education, University of Sfax, Sfax, Tunisia; ^2^Institute of Sport and Sport Science, Heidelberg University, Heidelberg, Germany; ^3^Centre de Recherches sur le Sport et le Mouvement (CeRSM EA 2931), Université Paris Nanterre, Nanterre, France; ^4^National Observatory of Sport, Tunis, Tunisia; ^5^Integrative and Experimental Training Science, Institute for Sport Sciences, University of Würzburg, Würzburg, Germany

**Keywords:** circadian rhythm, repeated sprint running, repeated exercise, mood, fatigue

## Abstract

**Objectives:** The aim of this study was to examine the effect of time of day on short-term repetitive maximal performance and psychological variables in elite judo athletes.

**Methods:** Fourteen Tunisian elite male judokas (age: 21 ± 1 years, height:172 ± 7 cm, body-mass: 70.0 ± 8.1 kg) performed a repeated shuttle sprint and jump ability (RSSJA) test (6 m × 2 m × 12.5 m every 25-s incorporating one countermovement jump (CMJ) between sprints) in the morning (7:00 a.m.) and afternoon (5:00 p.m.). Psychological variables (Profile of mood states (POMS-f) and Hooper questionnaires) were assessed before and ratings of perceived exertion (RPE) immediately after the RSSJA.

**Results:** Sprint times (*p* > 0.05) of the six repetition, fatigue index of sprints (*p* > 0.05) as well as mean (*p* > 0.05) jump height and fatigue index (*p* > 0.05) of CMJ did not differ between morning and afternoon. No differences were observed between the two times-of-day for anxiety, anger, confusion, depression, fatigue, interpersonal relationship, sleep, and muscle soreness (*p* > 0.05). Jump height in CMJ 3 and 4 (*p* < 0.05) and RPE (*p* < 0.05) and vigor (*p* < 0.01) scores were higher in the afternoon compared to the morning. Stress was higher in the morning compared to the afternoon (*p* < 0.01).

**Conclusion:** In contrast to previous research, repeated sprint running performance and mood states of the tested elite athletes showed no-strong dependency of time-of-day of testing. A possible explanation can be the habituation of the judo athletes to work out early in the morning.

## Introduction

Previous studies confirmed that physiological, psychological, and performance parameters during physical exercise present diurnal variations (Chtourou et al., 2013b, 2015; López-Samanes et al., 2017; Lopes-Silva et al., 2018; Pullinger et al., 2018). A recent review, revealed that performance in short-term high intensity exercise is time-of-day dependent with peak values were observed in the afternoon and amplitudes (i.e., difference between the maximal and minimal value) of ∼2–22% (Chtourou and Souissi, 2012). For example, in physical active students, peak and mean power during a Wingate test are significantly higher in the afternoon (5:00 p.m.) compared to the morning (7:00 a.m.) (Chtourou et al., 2011, 2012a). Likewise, it was shown that, jump height in squat jump and the countermovement jump (CMJ) are significantly higher in the afternoon (5:00 p.m.) compared to the morning (7:00 a.m.) (Chtourou et al., 2011, 2012a).

During repeated short-term high intensity efforts (i.e., effort required both aerobic and anaerobic pathways), previous studies confirmed the existence of a diurnal variation of performance only during the 1–3 first repetitions of the exercise (Racinais et al., 2005; Hammouda et al., 2011; Lopes-Silva et al., 2018; Pullinger et al., 2018). In this context, during 5 × 6-s cycling sprints with a 24-s recovery period, peak power was significantly higher in the afternoon (5:00 p.m.) than in the morning (7:00 a.m.) only during the first two sprints with amplitudes of 3.1–4.8 % (Chtourou et al., 2012b). The suppression of the diurnal variation of peak power during the three last sprints could be explained by a higher decrease of performance in the afternoon compared to the morning and to a greater utilization of the aerobic pathway during the last three repetitions of the exercise.

During exercise requiring higher energy production from the aerobic pathway, studies concerning performances (e.g., time trial cycling or covered distance during the Yo–Yo Test) and physiological responses (e.g., maximal oxygen uptake (VO_2max_), heart rate) to long-duration exercises yielded inconclusive results (see Chtourou and Souissi, 2012). As reviewed by Chtourou and Souissi (2012), it has been shown that VO_2max_ and the covered distance during the Yo–Yo test are time-of-day dependent with slight amplitudes of 1–3%. Thus, the diurnal variation of repeated short-term maximal performance could be reduced and/or suppressed while advancing in the exercise.

Reviewed data concerning the diurnal variation of muscle fatigue appear to be equivocal (Chtourou et al., 2013a). In this context, tennis performance (e.g., serving velocity and serving accuracy) is lower in the morning hours compared to the afternoon (López-Samanes et al., 2017). However, the majority of the studies reported that muscle fatigue with different physical tasks (e.g., Wingate test, repeated maximal voluntary contractions) is higher in the afternoon compared to the morning (Nicolas et al., 2007; Chtourou et al., 2012b, 2013a). Also, the subjective estimation of fatigue (i.e., rating of perceived exertion (RPE), Profile of Mood States (POMS) questionnaire) is higher in the afternoon compared to the morning (Chtourou et al., 2012b; Hammouda et al., 2012; Ammar et al., 2015).

In an attempt to explain the diurnal variation of exercise performances and fatigue, previous studies examined the effect of time-of-day on mood states (Chtourou et al., 2014; Hill and Hill, 2016). Sedentary subjects rated all aspects of negative mood (i.e., tension, depression, anger, fatigue, and confusion) and the total mood disturbance higher in the morning compared to the afternoon (Hill and Hill, 2016). In contrast, vigor is higher in the afternoon than the morning. However, prepubertal boys reported no-difference between the morning and the afternoon for both vigor and negative aspects of mood (Chtourou et al., 2014).

Otherwise, judo is a combat sport characterized by repetitive short-term high intensity efforts (Hamdi et al., 2013). During international competitions, judo athletes participate in 3–7 combats scheduled in the morning and the afternoon. Therefore, it appears that the investigation of the diurnal variation of repetitive short-term maximal performance, fatigue, and psychological variables of elite judokas is important for coaches and athletes. In fact, judo athletes in international events may compete at different time-of-day. Moreover, as judo athletes participating in international competitions complete three to ten training sessions per week with two to three sessions per day, it is important to examine the diurnal variations of mood (i.e., positive and negative mood states estimated by the POMS-f questionnaire), stress, fatigue, sleep, and muscle soreness (i.e., estimated by the Hooper questionnaire).

In view of the above considerations, the aim of the present study was to examine the effect of time-of-day on short-term repetitive maximal performance as well as mood, fatigue, stress, sleep, and muscle soreness in elite judo athletes.

As the results of Atkinson et al. (1993) showed that the amplitude of the diurnal variation of performance is reduced when the level of physical activity of the participant increased, we hypothesized that repeated sprint and jump performance, psychological variables (i.e., fatigue, stress, and mood states), sleep and muscle soreness of elite judo athletes were not strongly affected by the time-of-day of testing.

## Materials and Methods

### Participants

The required sample size was calculated using the software G^∗^Power (Faul et al., 2007) with alpha and power of 0.05 and 0.80, respectively. Based on the results of Chtourou et al. (2012c) and after discussions between the authors of the present study, effect sizes were fixed on 0.7 (large effect). To reach the desired power, at minimum eleven participants should be recruited in the present study. However, due to possible drop out of some participants, fifteen elite judo athletes were targeted for the present study. Due to a sensation of pain during the first session, one participant dropped out from the study. Fourteen healthy elite male judo athletes (age: 21 ± 1 years, height: 172 ± 7 cm, body-mass: 70.0 ± 8.1 kg) voluntarily participated and completed the experimental procedure of the present study.

The inclusion criteria for the present study were:

*(i)* All participants were elite athletes and were accustomed to a training workload of more than six training sessions (i.e., the duration of the training session was between 90 and 120 min) per week and had been frequently involved in Judo competitions.
*(ii)* All judo athletes were members of the Tunisian national Judo team.
*(iii)* Rae et al. (2015) recommended that the habitual time-of-day of training and the chronotype of the participants should be taken into account when examining the diurnal variation of performance. The training sessions of the participants were habitually scheduled in the morning and the afternoon hours. They, also, had standard times for sleeping habits (22 h00 min ± 1 h30 min). To have a group without “extreme type (extreme morning type or extreme evening type),” participants were selected as “neither type” based on their answers to the Horne and Östberg self-assessment questionnaire (Horne and Ostberg, 1976).

The study was conducted in a normal in-season training period with no-specific stress to participate to a major competition. A full description, written as well as orally, of the study protocol and the potential risks and benefits of the procedures involved was provided for the athletes. Subsequently, each participant signed a written informed consent form before participating. Participants were free to withdraw from the study at any time without further consequences. The study protocol complied with the Code of Ethics for human experimentation of the World Medical Association, the Declaration of Helsinki (World Medical Association [WMA], 2013). The experimental protocol was pre-approved by the ethical review board of the Institute.

### Experimental Design

After two familiarization sessions, participants performed the repeated shuttle sprint and jump ability (RSSJA) test according to the protocol proposed by Buchheit et al. (2010) in the morning at 7:00 a.m. and the afternoon at 5:00 p.m. in a randomized and counter-balance order. A recovery period of at least 36 h was insured between two successive sessions. The RSSJA test was performed following 15-min of standardized warm-up. Immediately after the test, participants rated their perceived exertion with the CR-10 RPE scale. The Hooper and the POMS-f questionnaires were completed before each test session.

To minimize confounding factors, participants were requested to keep their usual sleeping habits (i.e., 7 h ± 1 h sleep per night) during the experimental period. Before the morning tests, they were asked to wake-up at 6:00 a.m. and to come to the laboratory at 7:00 a.m. in a fasting state with consummation of one glass of water (∼15–20 cl), as recommended previously (Bougard et al., 2009). Before the afternoon tests, they had the same standard isocaloric meal at noon. After that meal, only water was allowed *ad libitum*.

### Repeated Sprint Test

All RSSJA tests were performed indoors on a PVC running surface with the participants wearing the same shoes and performing a split start each time. The RSSJA test consisted of six 2 × 12.5 m shuttle sprints with changes of direction of 180° every 25-s (∼5-s sprint and ∼20-s recovery) completed with maximum voluntary effort (Buchheit et al., 2010). During the recovery period, participants had to decelerate over a distance of 10 m, perform a CMJ (with hands on the hip), and then walked 36 m. They were verbally encouraged during the test to sprint and jump with all-out effort. Single beam timing lights (Wireless Timing Radio Controlled, Brower Timing System, Colorado, CO, United States) registered the time of each sprint. Timing light height was 1.0 m (i.e., targeting approximately hip height) and the starting line was placed 0.5 m in front of the initial timing light. The Optojump system (Microgate, Bolzano, Italy) was used to measure the jump height for each CMJ. The Optojump is a valid and reliable devise for the assessments of vertical jump height (ICCs = 0.98) (Glatthorn et al., 2011; Castagna et al., 2013).

The Mean and ideal time as well as fatigue index (s) were recorded during the RSSJA test whereas the ideal time and fatigue index was calculated as: Ideal time = best time (s) × number of sprints; and fatigue index = total time (s) - ideal time (s).

Likewise, mean and ideal height and the fatigue index were recorded from the six CMJ. With the ideal height = best height (cm) × number of CMJ and the fatigue index = ideal height (cm) - total height (cm).

### POMS-f Questionnaire

Profile of Mood States (POMS; McNair et al., 1971; French adaptation by Cayrou et al., 2000, 2003) was introduced in the study to collect data about the mood (i.e., their actual or “right now” mood) of participants just before they complete their test sessions. The questionnaire takes approximately 3 min to administer in a paper and pencil form and the raw scores for each item were analyzed. The POMS-f (French version) is a series of 65-adjectives measuring different emotional conditions (i.e., five negative moods: tension-anxiety, depression, anger-hostility, vigor-activity, fatigue, and confusion-bewilderment; one positive mood: vigor; and interpersonal relationships) and a total mood disturbances score (TMD) for which all scales except interpersonal relationships are taken into account [TMD = (Tension + Depression + Anger + Fatigue + Confusion) - Vigor]. Five-point Likert scales from 0 (i.e., indicate “not at all”) to 4 (i.e., indicate “extreme”), in relation to the context, are used. Psychometric evaluation of POMS-f has revealed satisfying qualities with high internal consistency estimates (0.82 < Cronbach’s alphas < 0.92) among both subscales and good test–retest (2 weeks) reliability ranges from 0.66 to 0.83 (Cayrou et al., 2000, 2003).

### RPE Scale and Hooper Questionnaire

Immediately after the RSSJA, each player’s RPE score was collected to ensure that the perceived effort was referred to the physical task only. The administrated revised category-ratio scale of Borg (Borg CR10 scales) allows participants to give a subjective exertion rating for the physical task. This RPE scale runs from 0 (nothing at all; indicate how you feel when sitting in a chair) to 10 (very, very hard; indicate how you feel at the end of a very, very difficult activity). The Borg CR10 version is a reliable indicator of physical discomfort (i.e., good test–retest reliability coefficients: 0.50 < *r* < 0.85), has sound psychometric properties and is strongly correlated with several other physiological measures of exertion (Borg, 1982; Impellizzeri et al., 2004).

Concerning the Hooper questionnaire, 15 min before each experimental session, participants were asked to rate their subjective estimation of the quality of the prior night sleep as well as fatigue, stress, and muscle soreness (DOMS) levels. This strategy allows detecting individual signs of pre-fatigue (Hooper et al., 1995). The four subjective ratings were recorded on a scale ranging from 1 to 7, with 1 indicate very, very good for sleep quality and very, very low for stress, fatigue, and DOMS and 7 indicate very, very bad for sleep quality and very, very high for stress, fatigue, and DOMS (Hooper and Mackinnon, 1995).

For both psychological scales, all judokas had been familiarized to these scales for at least 1 month before the start of the study and followed standardized instructions for rating-session collection.

### Statistical Analysis

Data were analyzed using the Statistica software (StatSoft, France; version 10). The measured parameters were presented as mean ± SD. The Shapiro–Wilk test verified the normality of the distribution. Data of the six sprints of the RSSJA test were not normally distributed. Therefore, we compared the morning and afternoon performances of the six sprints of the RSSJA test using the Wilcoxon test. Data of the six CMJ, the mean and ideal height as well as fatigue index during the six repetitions of the CMJ and anxiety, anger, confusion, interpersonal relationship, and TMD registered by the POMS-f questionnaire were analyzed using Student’s *t*-test after confirming normal distribution. RPE scores, data of the Hooper questionnaire and depression, fatigue and vigor registered by the POMS-f questionnaire were analyzed using the Wilcoxon test as the data were not normally distributed. The level of statistical significance was set at *p* < 0.05.

## Results

### Repeated Sprint Performances

The sprint times of each of the six sprints recorded in the morning and the afternoon did not differ statistically between the two times of testing (Table 1). Likewise, there are no-significant differences between the two times-of-day for mean and ideal time as well as the fatigue index recorded during the RSSJA test (Table 1).

**Table 1:** Six sprint times, mean, and ideal times as well as fatigue index recorded in the morning and the afternoon during the repeated shuttle-sprint and jump ability test.

	Morning	Afternoon	*p*
Sprint 1 (s)	5.26 ± 0.22	5.23 ± 0.24	0.609
Sprint 2 (s)	5.30 ± 0.20	5.31 ± 0.23	0.711
Sprint 3 (s)	5.37 ± 0.21	5.37 ± 0.25	0.954
Sprint 4 (s)	5.45 ± 0.26	5.43 ± 0.26	0.477
Sprint 5 (s)	5.53 ± 0.26	5.52 ± 0.28	0.280
Sprint 6 (s)	5.62 ± 0.25	5.60 ± 0.24	0.470
Mean time (s)	5.42 ± 0.23	5.41 ± 0.24	0.514
Ideal time (s)	31.55 ± 1.29	31.37 ± 1.46	0.609
Fatigue index (s)	0.99 ± 0.35	1.08 ± 0.45	0.570


*The values presented are means ± SD. s, second.*

### Repeated CMJ Performances

The *t*-test revealed that CMJ jump height 3 and 4 were significantly higher in the afternoon compared to the morning (CMJ 3: *t* = 3.12, *p* < 0.01; CMJ 4: *t* = 2.52, *p* < 0.05) (Table 2). However, there were no-significant differences between the two times of testing for the CMJ 2, 5, and 6.

**Table 2:** Six counter movement jump (CMJ) heights, mean and ideal jump heights as well as fatigue index recorded in the morning and the afternoon during the counter-movement jump in the repeated shuttle-sprint and jump ability test.

	Morning	Afternoon	*p*
CMJ 1 (cm)	29.86 ± 2.91	31.09 ± 3.29	0.090
CMJ 2 (cm)	29.05 ± 2.52	30.01 ± 3.40	0.153
CMJ 3 (cm)	27.87 ± 2.66	29.41 ± 3.33	0.007
CMJ 4 (cm)	27.25 ± 2.63	28.62 ± 3.30	0.024
CMJ 5 (cm)	26.58 ± 2.47	27.26 ± 3.38	0.259
CMJ 6 (cm)	25.27 ± 1.90	26.17 ± 3.81	0.236
Mean height (cm)	27.65 ± 2.40	28.76 ± 3.33	0.057
Ideal height (cm)	179.16 ± 17.44	186.56 ± 19.74	0.091
Fatigue index (cm)	13.29 ± 5.76	13.99 ± 5.90	0.751


*The values presented are means ± SD. cm, centimeter.*

The mean height of all six CMJ were not significantly higher in the afternoon compared to the morning (*p* > 0.05) (Table 2). Accordingly, no-significant differences between the two times of testing were evident for the ideal height and the fatigue index (Table 2).

### RPE Scores

The RPE scores were significantly higher in the afternoon compared the morning (6.47 ± 1.06 a.u. vs. 5.93 ± 1.10 a.u.; *p* < 0.05).

### Hooper Questionnaire

Stress was significantly higher in the morning compared to the afternoon (*p* < 0.05) (Table 3). However, no-significant differences between the two times-of-day were observed for fatigue, sleep, and muscle soreness.

**Table 3:** Mean ( ± SD) fatigue, stress, sleep, and muscle soreness recorded in the morning and the afternoon by the Hooper questionnaire.

	Morning	Afternoon	*p*
Fatigue (a.u.)	3.1 ± 0.9	3.2 ± 0.9	0.814
Stress (a.u.)	3.0 ± 1.1	2.1 ± 0.8	0.018 ^∗^
Sleep (a.u.)	2.6 ± 1.1	2.4 ± 1.0	0.477
Muscle soreness (a.u.)	2.2 ± 0.9	2.3 ± 1.2	0.594


*The values presented are means ± SD. ^∗^ significant difference for comparison of the time-of-day; a.u., arbitrary unit.*

### Profile of Mood States Questionnaire

Vigor was significantly higher in the afternoon compared to the morning (*p* < 0.05) (Table 4). However, no-significant differences between the two times-of-day were registered for anxiety, anger, confusion, depression, fatigue, interpersonal relationship and TMD (Table 4).

**Table 4:** Mean ( ± SD) anxiety, anger, confusion, depression, fatigue, vigor, interpersonal relationship, and total mood disturbances (TMD) scores registered by the Profile of Mood State questionnaire in the morning and the afternoon.

	Morning	Afternoon	*p*
Anxiety (a.u.)	8.9 ± 6.2	8.8 ± 4.5	0.956
Anger (a.u.)	8.8 ± 5.7	9.5 ± 5.3	0.614
Confusion (a.u.)	8.0 ± 4.4	7.1 ± 4.5	0.365
Depression (a.u.)	6.0 ± 7.3	5.1 ± 4.6	0.695
Fatigue (a.u.)	6.4 ± 5.6	6.3 ± 5.1	0.706
Vigor (a.u.)	19.8 ± 5.1	23.7 ± 4.2	0.019 ^∗^
Interpersonal relationship (a.u.)	18.0 ± 4.3	19.3 ± 4.3	0.309
Emotional distress scores (a.u.)	18.3 ± 26.4	13.1 ± 20.5	0.330


*The values presented are means ± SD. ^∗^ significant difference for comparison of the time-of-day; a.u., arbitrary unit.*

## Discussion

The main findings of the present study were as follows: (*i*) no morning-afternoon differences for repeated sprint performances, negative mood states as well as for fatigue, sleep, and muscle soreness; (*ii*) stress estimated by the Hooper questionnaire was higher in the morning; (*iii*) jump height in the 3rd and the 4th jump of CMJ were higher in the afternoon; (*iii*) RPE and vigor estimated by the POMS-f questionnaire were higher in the afternoon.

In contrast to previous findings (Racinais et al., 2005; Hammouda et al., 2011; Chtourou et al., 2012b) the repeated sprint performances in the present study was not different between the morning and afternoon. The discrepancies between the present study’ findings and those of Racinais et al. (2005); Hammouda et al. (2011) and Chtourou et al. (2012b) could be related to: (*i*) differences in the involved muscle groups (i.e., cycling in the previous studies vs. sprint running and jumping in the present study) (Glaister et al., 2008) and (*ii*) the training level of the participants (i.e., the amplitude of the circadian rhythm of muscle strength was reduced with the improvement of the training level of the participants) (Atkinson et al., 1993). Atkinson et al. (1993) compared the time-of-day effects on performances between sedentary and physically active participants and revealed greater amplitudes (i.e., morning to afternoon differences) in trained participants. However, to the best of our knowledge, no-previous study compared physically active and well-trained individuals in terms of the effect of time-of day on performance parameters.

In well trained judo athletes (Chtourou et al., 2013a) and tennis players (López-Samanes et al., 2017) significant time-of-day differences have been reported, demonstrating diminished performances in the morning [i.e., handgrip (Chtourou et al., 2013a; López-Samanes et al., 2017), power during a Wingate test (Chtourou et al., 2013a) and CMJ, 10-m run, agility sprint, serving velocity and accuracy (López-Samanes et al., 2017)]. The divergence between the present study and other findings (Chtourou et al., 2013a; López-Samanes et al., 2017) could be attributed to the time-of-day of regular training of athletes. In fact, the judo athletes recruited for the present study trained two times per day with a morning training session (i.e., fitness session) from 7:00 to 8:00 a.m. and an afternoon training session (i.e., technical-tactical session) at 4:00–6:00 p.m. In this context, previous studies (Chtourou et al., 2012a,c) reported that regular training in the morning hours could remove or even inverse the diurnal variation of short-term maximal performances. Furthermore, the duration of the warm-up (15 min) applied in the present study could explain the non-significant difference between the two times-of-day for repeated sprinting. Indeed, Souissi et al. (2010) indicated that the amplitude of the diurnal variation of short-term maximal performance was reduced with a 15 min warm-up in comparison to a 5 min warm-up.

On the other hand, in agreement with previous studies (Chtourou et al., 2012c,d; López-Samanes et al., 2017; Mhenni et al., 2017), the present study confirmed that CMJ performance during the 3rd and the 4th repetitions was greater in the afternoon compared to the morning. Also, the present study did not reveal significant differences between 7:00 a.m. and 5:00 p.m. for negative mood. The improved CMJ performance in the afternoon could be explained in part by the better vigor scores observed in the afternoon compared to the morning. Indeed, vigor estimated by the POMS-f questionnaire was better in the afternoon compared to the morning. Although the present study did not report a significant difference between morning and afternoon for fatigue, sleep and muscle soreness estimated by the Hooper questionnaire, the item “stress,” measured by the Hooper questionnaire, was significantly higher in the morning compared to the afternoon. This could partially explain the impaired performance observed at this time-of-day.

However, concerning fatigue calculated for both the repetition of sprints or CMJs, no time-of-day effects were observed. Likewise, subjective fatigue recorded by the POMS-f and the Hooper questionnaires were not different between the morning and the afternoon. Therefore, the present study did not confirm the hypothesis that muscle fatigue is higher in the afternoon. However, the RPE scores were elevated in the afternoon compared to the morning. These results are in line with previous results regarding repeated cycling exercise (Hammouda et al., 2011).

### Limitations

In the present study, sprint running times were measured with single beam timing lights. It had been shown that single beam timing lights can be triggered early by lifted upper or lower extremities (Altmann et al., 2015; Haugen and Buchheit, 2016). The associated measurement error can be considerably high and up to approximately 10% of absolute sprint time in 5 m sprints compared to high-speed video timing, but the relative measurement error decreases with the length of the sprint distance (Altmann et al., 2015, 2017). For 30 m sprints, the relative measurement error is 1.5–2.5 % of absolute sprint time (Altmann et al., 2017).

Another limitation for the present study is that the selected performance tests are not specific to judo (i.e., neither the task nor the duration). Indeed, Franchini et al. (2013) reported that the time structure of a judo combat is of 20–30-s of fight and 10-s of break. Also, it has been indicated that, in judo competition, both upper and lower body muscle groups are involved Pocecco et al. (2012).

## Conclusion

The results reveal that, in elite judo athletes, neither repeated sprint-running performance nor mood or items of the Hooper questionnaire showed a strong dependency on time-of-day. However, vigor and RPE scores were higher and stress scores were lower in the afternoon compared to the morning. Of performance parameters, solely jump height in CMJ1, 3 and 4 was elevated in the afternoon compared to the morning. However, fatigue indices of repeated sprinting and repeated jumping showed no-differences between the morning and afternoon. The lacking presence of circadian variations of sprint running performance, fatigue indices and the majority of psychological parameters in the present study could be explained by the habituation of participants to early morning training hours.

However, since jump height in repeated CMJ is not directly or indirect related to judo combat performance it may not have an important impact in the outcome of a judo combats in professional judokas.

Indeed, the lack of strong diurnal variations of performance and fatigue during repeated high-intensity efforts, as well as the independency of majority of psychological parameters in the context of such intensive exercise demonstrate that, for elite athletes, the time-of-day for short-term maximal repetitive exercise should not be considered necessarily.

On the other hand, future research should evaluate if performance during repeated high-intensity bouts using a specific judo test is impaired in the morning compared to the afternoon.

## Author Contributions

HC, HsF, HzF, OH, and NS conceived the idea of conducting the present study and determined the study design and executed the experimental sessions. HC, FE, and BS computed and analyzed the data and wrote the manuscript. BS and FE performed extensive proofreading of the manuscript and helped to supervise the project. All authors discussed the results as well as the final version of the manuscript and contributed to the final version of the manuscript.

## Conflict of Interest Statement

The authors declare that the research was conducted in the absence of any commercial or financial relationships that could be construed as a potential conflict of interest.

## References

[B1] Altmann S. Hoffmann M. Kurz G. Neumann R. Woll A. Haertel S. (2015). Different starting distances affect 5-m sprint times. *J. Strength Cond. Res.* 29 2361–2366. 10.1519/JSC.0000000000000865 25647648

[B2] Altmann S. Spielmann M. Engel F. A. Neumann R. Ringhof S. Oriwol D. (2017). Validity of single-beam timing lights at different heights. *J. Strength Cond. Res.* 31 1994–1999. 10.1519/JSC.0000000000001889 28277431

[B3] Ammar A. Chtourou H. Trabelsi K. Padulo J. Turki M. El Abed K. (2015). Temporal specificity of training: intra-day effects on biochemical responses and Olympic-Weightlifting performances. *J. Sports Sci.* 33 358–368. 10.1080/02640414.2014.944559 25117722

[B4] Atkinson G. Coldwells A. Reilly T. Waterhouse J. (1993). A comparison of circadian rhythms in work performance between physically active and inactive subjects. *Ergonomics* 36 273–281. 10.1080/00140139308967882 8440222

[B5] Borg G. A. (1982). Psychophysical bases of perceived exertion. *Med. Sci. Sports Exerc.* 14 377–381. 10.1249/00005768-198205000-00012 7154893

[B6] Bougard C. Bessot N. Moussay S. Sesboue B. Gauthier A. (2009). Effects of waking time and breakfast intake prior to evaluation of physical performance in the early morning. *Chronobiol. Int.* 26 307–323. 10.1080/07420520902774532 19212843

[B7] Buchheit M. Spencer M. Ahmaidi S. (2010). Reliability, usefulness, and validity of a repeated sprint and jump ability test. *Int. J. Sports Physiol. Perform.* 5 3–17. 10.1123/ijspp.5.1.3 20308692

[B8] Castagna C. Ganzetti M. Ditroilo M. Giovannelli M. Rocchetti A. Manzi V. (2013). Concurrent validity of vertical jump performance assessment systems. *J. Strength Cond. Res.* 27 761–768. 10.1519/JSC.0b013e31825dbcc5 22648140

[B9] Cayrou S. Dickès P. Dolbeault S. (2003). Version française du profile of mood states (POMS-f) [French version of the Profile Of Mood States (POMS-f)]. *J. Ther. Comport. Cogn.* 13 83–88.

[B10] Cayrou S. Dickès P. Gauvin-Piquard A. Dolbeault S. Callahan S. Rogé B. (2000). Validation de la traduction française du POMS (profile of mood states). *Psychol. Psychométr.* 21 5–22.

[B11] Chtourou H. Aloui A. Hammouda O. Chaouachi A. Chamari K. Souissi N. (2013a). The effect of time-of-day and judo match on short-term maximal performances in judokas. *Biol. Rhythm Res.* 44 797–806. 10.1080/09291016.2012.756269 23974210

[B12] Chtourou H. Aloui A. Hammouda O. Souissi N. Chaouachi A. (2014). Diurnal variation in long- and short-duration exercise performance and mood states in boys. *Sport Sci. Health* 10 183–187. 10.1007/s11332-014-0190-0

[B13] Chtourou H. Ammar A. Nikolaidis P. T. Abdel Karim O. Souissi N. Chamari K. (2015). Post-resistance training detraining: time-of-day effects on training and testing outcomes. *Biol. Rhythm Res.* 46 897–907. 10.1080/09291016.2015.1063204

[B14] Chtourou H. Hammouda O. Aloui A. Souissi N. (2013b). Effect of time-of-day on muscle fatigue: a review. *J. Nov. Physiother.* 3:160 10.4172/2165-7025.1000160

[B15] Chtourou H. Hammouda O. Chaouachi A. Chamari K. Souissi N. (2012a). The effect of time-of-day and Ramadan fasting on anaerobic performances. *Int. J. Sports Med.* 33 142–147. 10.1055/s-0031-1286251 22318530

[B16] Chtourou H. Hammouda O. Souissi H. Chamari K. Chaouachi A. Souissi N. (2012b). Diurnal variations in physical performances related to football in young soccer players. *Asian J. Sports Med.* 3 139–144. 23012632 10.5812/asjsm.34604 PMC3445640

[B17] Chtourou H. Driss T. Souissi S. Gam A. Chaouachi A. Souissi N. (2012c). The effect of strength training at the same time of the day on the diurnal fluctuations of muscular anaerobic performances. *J. Strength Cond. Res.* 26 217–225. 10.1519/JSC.0b013e31821d5e8d 21993020

[B18] Chtourou H. Chaouachi A. Driss T. Dogui M. Behm D. G. Chamari K. (2012d). The effect of training at the same time of day and tapering period on the diurnal variation of short exercise performances. *J. Strength Cond. Res.* 26 697–708. 10.1519/JSC.0b013e3182281c87 21857363

[B19] Chtourou H. Souissi N. (2012). The effect of training at a specific time of day: a review. *J. Strength Cond. Res.* 26 1984–2005. 10.1519/JSC.0b013e31825770a7 22531613

[B20] Chtourou H. Zarrouk N. Chaouachi A. Dogui M. Behm D. G. Chamari K. (2011). Diurnal variation in Wingate-test performance and associated electromyographic parameters. *Chronobiol. Int.* 28 706–713. 10.3109/07420528.2011.596295 21793694

[B21] Faul F. Erdfelder E. Lang A. G. Buchner A. (2007). G^∗^ Power 3: a flexible statistical power analysis program for the social, behavioral, and biomedical sciences. *Behav. Res. Methods* 39 175–191. 10.3758/BF03193146 17695343

[B22] Franchini E. Artioli G. G. Brito C. J. (2013). Judo combat: time-motion analysis and physiology. *Int. J. Perform. Anal. Sport* 13 624–641. 10.1080/24748668.2013.11868676

[B23] Glaister M. Howatson G. Pattison J. R. McInnes G. (2008). The reliability and validity of fatigue measures during multiple-sprint work: an issue revisited. *J. Strength Cond. Res.* 22 1597–1601. 10.1519/JSC.0b013e318181ab80 18714226

[B24] Glatthorn J. F. Gouge S. Nussbaumer S. Stauffacher S. Impellizzeri F. M. Maffiuletti N. A. (2011). Validity and reliability of Optojump photoelectric cells for estimating vertical jump height. *J. Strength Cond. Res.* 25 556–560. 10.1519/JSC.0b013e3181ccb18d 20647944

[B25] Hamdi C. Hana B. Asma A. Najla I. Liwa M. Karim C. (2013). Effects of recovery type on judokas’ short-term maximal performances during a simulated competition. *Br. J. Sports Med.* 47:e3 10.1136/bjsports-2013-092558.16 19636586

[B26] Hammouda O. Chtourou H. Chahed H. Ferchichi S. Chaouachi A. Kallel C. (2012). High intensity exercise affects diurnal variation of some biological markers in trained subjects. *Int. J. Sports Med.* 33 886–891. 10.1055/s-0032-1301887 22791622

[B27] Hammouda O. Chtourou H. Chahed H. Ferchichi S. Kallel C. Miled A. (2011). Diurnal variations of plasma homocysteine, total antioxidant status, and biological markers of muscle injury during repeated sprint: effect on performance and muscle fatigue–a pilot study. *Chronobiol. Int.* 28 958–967. 10.3109/07420528.2011.613683 22080741

[B28] Haugen T. Buchheit M. (2016). Sprint running performance monitoring: methodological and practical considerations. *Sports Med.* 46 641–656. 10.1007/s40279-015-0446-0 26660758

[B29] Hill C. M. Hill D. W. (2016). Influence of time of day on responses to the profile of mood states. *Percept. Mot. Skills* 72:434 10.2466/pms.1991.72.2.434

[B30] Hooper S. L. Mackinnon L. T. (1995). Monitoring overtraining in athletes. Recommendations. *Sports Med.* 20 321–327. 10.2165/00007256-199520050-00003 8571005

[B31] Hooper S. L. Mackinnon L. T. Howard A. Gordon R. D. Bachmann A. W. (1995). Markers for monitoring overtraining and recovery. *Med. Sci. Sports Exerc.* 27 106–112. 10.1249/00005768-199501000-00019 7898325

[B32] Horne J. A. Ostberg O. (1976). A self-assessment questionnaire to determine morningness-eveningness in human circadian rhythms. *Int. J. Chronobiol.* 4 97–110. 1027738

[B33] Impellizzeri F. M. Rampinini E. Coutts A. J. Sassi A. Marcora S. M. (2004). Use of RPE-based training load in soccer. *Med. Sci. Sports Exerc.* 36 1042–1047. 10.1249/01.MSS.0000128199.23901.2F 15179175

[B34] Lopes-Silva J. P. Santos J. F. D. S. Franchini E. (2018). Can caffeine supplementation reverse the effect of time of day on repeated sprint exercise performance? *Appl. Physiol. Nutr. Metab.* 10.1139/apnm-2018-0373 [Epub ahead of print]. 30058345

[B35] López-Samanes Á Moreno-Pérez D. Maté-Muñoz J. L. Domínguez R. Pallarés J. G. Mora-Rodriguez R. (2017). Circadian rhythm effect on physical tennis performance in trained male players. *J. Sports Sci.* 35 2121–2128. 10.1080/02640414.2016.1258481 27918240

[B36] McNair D. Lorr M. Droppleman L. (1971). *EDITS Manual for the Profile of Mood States.* San Diego, CA: Editorial and Industrial Testing Service.

[B37] Mhenni T. Michalsik L. B. Mejri M. A. Yousfi N. Chaouachi A. Souissi N. (2017). Morning-evening difference of team-handball-related short-term maximal physical performances in female team handball players. *J. Sports Sci.* 35 912–920. 10.1080/02640414.2016.1201212 27352917

[B38] Nicolas A. Gauthier A. Michaut A. Davenne D. (2007). Effect of circadian rhythm of neuromuscular properties on muscle fatigue during concentric and eccentric isokinetic actions. *Isokinet. Exerc. Sci.* 15 117–129.

[B39] Pocecco E. Gatterer H. Ruedl G. Burtscher M. (2012). Specific exercise testing in judo athletes. *Arch. Budo* 8 133–139. 10.12659/AOB.883246

[B40] Pullinger S. A. Oksa J. Clark L. F. Guyatt J. W. Newlove A. Burniston J. G. (2018). Diurnal variation in repeated sprint performance cannot be offset when rectal and muscle temperatures are at optimal levels (38.5° C). *Chronobiol. Int.* 35 1054–1065. 10.1080/07420528.2018.1454938 29566344

[B41] Racinais S. Connes P. Bishop D. Blonc S. Hue O. (2005). Morning versus evening power output and repeated-sprint ability. *Chronobiol. Int.* 22 1029–1039. 10.1080/07420520500397918 16393706

[B42] Rae D. E. Stephenson K. J. Roden L. C. (2015). Factors to consider when assessing diurnal variation in sports performance: the influence of chronotype and habitual training time-of-day. *Eur. J. Appl. Physiol.* 115 1339–1349. 10.1007/s00421-015-3109-9 25631930

[B43] Souissi N. Driss T. Chamari K. Vandewalle H. Davenne D. Gam A. (2010). Diurnal variation in Wingate test performances: influence of active warm-up. *Chronobiol. Int.* 27 640–652. 10.3109/07420528.2010.483157 20524806

[B44] World Medical Association [WMA] (2013). Declaration of Helsinki: ethical principles for medical research involving human subjects. *JAMA* 310 2191–2194. 10.1001/jama.2013.281053 24141714

